# Nischarin Deletion Reduces Oxidative Metabolism and Overall ATP: A Study Using a Novel *NISCH^Δ5-6^* Knockout Mouse Model

**DOI:** 10.3390/ijms23031374

**Published:** 2022-01-25

**Authors:** Tina H. Nguyen, Hassan Yousefi, Samuel C. Okpechi, Lothar Lauterboeck, Shengli Dong, Qinglin Yang, Suresh K. Alahari

**Affiliations:** 1 Department of Biochemistry and Molecular Biology, Louisiana State University Health Science Center, New Orleans, LA 70112, USA; tngu85@lsuhsc.edu (T.H.N.); hyouse@lsuhsc.edu (H.Y.); sokpec@lsuhsc.edu (S.C.O.); Shengli.Dong@tykmedicines.com (S.D.); 2 Cardiovascular Center of Excellence, School of Medicine, Louisiana State University Health Science Center, New Orleans, LA 70112, USA; llaute@lsuhsc.edu (L.L.); qyang1@lsuhsc.edu (Q.Y.); 3 Department of Pharmacology, Louisiana State University Health Science Center, New Orleans, LA 70112, USA

**Keywords:** Nischarin, knockout mouse model, migration, metabolism

## Abstract

Nischarin (Nisch) is a cytosolic scaffolding protein that harbors tumor-suppressor-like characteristics. Previous studies have shown that Nisch functions as a scaffolding protein and regulates multiple biological activities. In the current study, we prepared a complete Nisch knockout model, for the first time, by deletion of exons 5 and 6. This knockout model was confirmed by Qrt–PCR and Western blotting with products from mouse embryonic fibroblast (MEF) cells. Embryos and adult mice of knockouts are significantly smaller than their wild-type counterparts. Deletion of Nisch enhanced cell migration, as demonstrated by wound type and transwell migration assays. Since the animals were small in size, we investigated Nisch’s effect on metabolism by conducting several assays using the Seahorse analyzer system. These data indicate that Nisch null cells have lower oxygen consumption rates, lower ATP production, and lower levels of proton leak. We examined the expression of 15 genes involved in lipid and fat metabolism, as well as cell growth, and noted a significant increase in expression for many genes in Nischarin null animals. In summary, our results show that Nischarin plays an important physiological role in metabolic homeostasis.

## 1. Introduction

Nischarin (Nisch) is a key player in several molecular pathways that regulate migration in both brain development and cancer progression [1,2]. The protein was named for this feature, which is a derivation of a classic Sanskrit term that signifies “slowness of motion” [3]. The gene that encodes Nisch is located on chromosome region 3p.21.1, which is a locus that is often lost or hypermethylated in several types of cancer [4,5]. When Nisch is either downregulated or silenced altogether, cancer cells tend to be more aggressive and invasive, as seen in the case of triple-negative breast cancer cells [6,7]. Nisch has also been found to play a role in metabolic homeostasis [8], which is a new area of interest in Nisch’s role in biology. In this study, we aim to characterize a Nisch-knockout mouse model in order to corroborate previous findings on the influence of Nisch on migration, as well as closely investigate the effects of Nisch on metabolic pathways.

Protein interactions with Nisch that regulate migration include those with PAK1, LIMK, and LKB1 [8]. Each of these proteins functions in the same pathway, responsible for actin filament assembly necessary for lamellipodia formation. Their collective interaction with Nisch indicates a multifaceted role in regulating migration. In the context of metabolism, cells from Nisch-mutated mice exhibit reduced anabolic protein expression and increased catabolic protein expression [8]. Without Nisch to regulate phosphorylation of AMPK, the CREB-regulated transcription coactivator 1 (CRTC1) protein is activated by AMPK activity and associates with the cAMP-response element-binding protein (CREB). The pair then translocates to the nucleus to mediate the transcription of gluconeogenic genes [9]. Similar to the regulation of focal adhesion and invasion proteins, the decrease in metabolic protein expression in these mutant mice is likely a downstream effect of Nisch interaction. It has been noted that with the extensive number of proteins that can bind to Nisch, as typical with scaffolding proteins, it is difficult to determine the signaling pathways that drive differential protein expression when Nisch is up- or downregulated.

We sought to generate a knockout mouse model for Nisch by deletion of exons 5 and 6. These exons do not harbor any motifs that suggest protein function; thus, we predict them to be involved in mRNA or protein stabilization. Currently, there are two mouse models for Nisch: Zhang et al. generated a knockout model through the deletion of exon 4, and Dong et al. generated a loss-of-function model through the deletion of exons 7–10 [10,11]. Using the model proposed in this paper, we investigated migration and metabolism in cells without the context of cancer. By better understanding how Nisch modulates cellular behavior, we can potentially translate our studies into the clinical setting with gene therapy.

## 2. Results

### 2.1. The Nisch^Δ5-6^ Mouse Is a Valid Knockout Model

**Figure 1:**
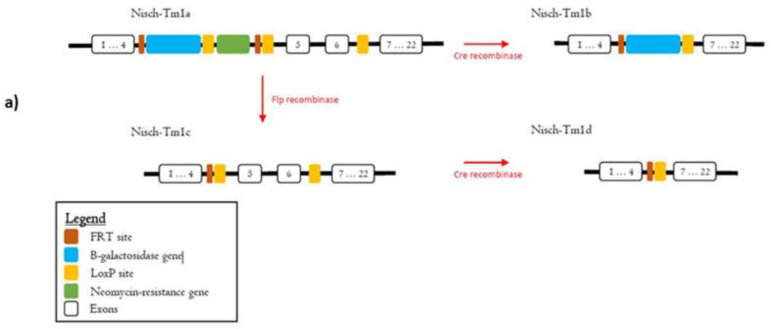

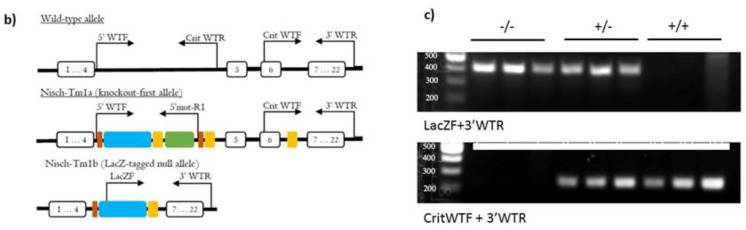
Knockout mice were generated using a reporter-tagged allele: (**a**) Nisch-Tm1a mice are the conditional knockout-first allele and can be crossed with either a Cre recombinase that recognizes LoxP sites or an Flp recombinase that recognizes FRT sites. The Cre recombinase cross yields Nisch-Tm1b mice, which have a LacZ-tagged null allele. The Flp recombinase cross yields Nisch-Tm1c mice, which have a conditional-ready allele. Nisch-Tm1c mice may be subsequently crossed with Cre recombinase to yield Nisch-Tm1d mice, which have a tissue-specific null allele; (**b**) Tm1b mice were genotyped using the primers shown in the figure. The wild-type allele can be identified by using either 5′ WTF and Crit WTR or Crit WTF and 3′WTR. These primers amplify regions surrounding exons 5 and 6, the targeted deletions for the knockout model. Mice with the conditional allele can be genotyped by the presence of a band when primers 5′ WTF and 5′mut-R1 were used for PCR. The introduction of the LacZ-containing cassette is distinguished from the wild-type PCR primer sets due to the product not being able to anneal after the extension period, secondary to the longer sequence. Finally, the knockout allele was detected by using primers LacZF and 3′WTR. The deletion of the exons 5 and 6 prevent primers that would otherwise detect the wild-type or Tm1a allele; (**c**) PCR products for primer set LacZF and 3′WTR yield a band at 400 bp, indicating the deletion of the critical region containing exons 5 and 6; PCR products for primer set Crit WTF and 3′WTR yield a band at 210 bp, indicating the wild-type allele. Lanes 1–3 (after the marker) contain DNA from Nisch^−/−^ MEF cells, lanes 4–6 contain DNA from Nisch^+/−^ MEF cells, and lanes 7–9 contain DNA from Nisch^+/+^ MEF cells. Experiments were performed in triplicate and repeated two times.

We obtained genetically modified mice from MRC Harwell containing a LacZ cassette and floxed sites surrounding exons 5 and 6 of Nisch. The knockout, first-reporter tagged insertion allele model allows for several breeding strategies, which can provide different models for further studying the function of Nisch. The presence of the LacZ gene in the Nisch-Tm1b mouse model serves as a reporter gene, through which we may confirm the expression of Nisch in selective tissues. A schematic of possible strategies is represented in Figure 1a. Nisch-tm1b mice were backcrossed onto the C57BL/6J background, in order to compare our results with previous studies [10]. Genomic DNA PCR was performed. A schematic of the PCR primers used to amplify the target PCR products are shown in Figure 1b. PCR products are shown in Figure 1c, where the knockout allele is about 400 bp long, and the wild-type allele is about 240 bp long.

**Figure 2:**
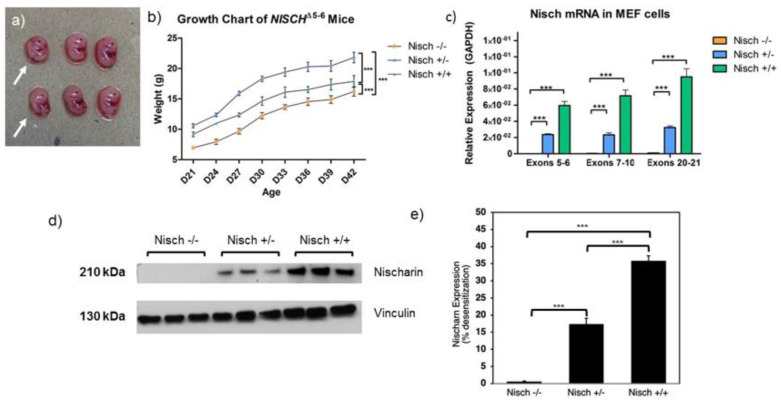
Knockout mouse phenotype shows attenuated growth. MEF cells were extracted as described in the Methods Section: (**a**) upon removal of the embryos from the amniotic sac, a slight difference in size is noted. The same phenotype has been shown in previous Nisch-mutated mouse models; (**b**) pups were measured for weight 3 weeks after weaning. Consistent with previous studies, Nisch^−/−^ mice exhibited smaller sizes. Nisch^+/−^ mice were significantly larger, compared with Nisch^+/+^ mice; (**c**) three regions of the Nisch gene were tested for quantification of mRNA in the MEF cells: exons 5–6, exons 7–10, and exons 20–21. Each target gene’s expression levels were normalized to GAPDH. Data shown represent the mean ± SEM from the three independent experiments, each performed in triplicates; (**d**) Nisch^−/−^, Nisch^+/−^, and Nisch^+/+^ cell lysates were obtained in triplicate repeats and run via SDS–PAGE for detection of Nischarin protein expression; (**e**) quantitation data of (**d**). Statistically significant values of *** *p* < 0001 were assigned using two-way *t*-test analysis between the three genotypes. Experiments were performed in triplicate and repeated two times.

We sought to generate mouse embryonic fibroblast (MEF) cells in order to study their cellular behavior. Since Nisch is widely studied in the context of cancer, we wanted to study fibroblasts for their complete mesenchymal-like character. We cultured three replicates of each Nisch^−/−^, Nisch^+/−^, and Nisch^+/+^ MEF cell population for RNA and protein analysis. Three sets of qRT–PCR primers were designed for the detection of different regions of Nisch to confirm the presence of mRNA. The first set detects exons 5 and 6, which was the deletion used in this mouse model. We also probed for expression of exons 7–10 and 20–21 to investigate whether or not there was any Nisch-encoding mRNA present in the cells. Nisch^−/−^ animals do not express Nischarin mRNA, as demonstrated with three different sets of primers (Figure 2c).

Since mRNA levels do not always correlate with protein expression, we also performed a Western blot to further confirm that the Nisch-tm1b mice were a true knockout model. Cell lysates were prepared using the same MEF cells from which the RNA was collected. We used an antibody that recognizes epitopes 1212–1504, preventing the possibility of failure of detecting Nisch secondary to mutations at the N terminus. As shown in Figure 2d,e, our results show no bands detected by the antibody, supporting our hypothesis that deletion of exons 5–6 yields a true knockout mouse for Nisch. To normalize protein levels, vinculin was used as the housekeeping protein. The data from the qRT–PCR and Western blots show a proportional decrease in Nisch mRNA and protein expression in the heterozygous MEF cell samples, compared with the wild-type MEF cell samples.

### 2.2. Genetically Modified Mice Exhibit a Small-Size Phenotype

Consistent with Nisch-mutated mice used in previous studies, *Nisch^Δ5-6^* mice display a smaller size and attenuated growth phenotype [8,10,11]. From the first day of birth, these mice are observed to be smaller than the wild-type and heterozygous mice, which are indistinguishable from each other. When a litter is born, it is possible to predict the number of Nisch^−/−^ mice, which can then be confirmed by genotyping once the mice are weaned. Even during the process of harvesting MEF cells, two embryos in Figure 2a are observably smaller than the rest of the embryos, as indicated by the arrows. We were able to confirm the prediction that these embryos were Nisch^−/−^ with PCR. To further investigate these differences in size and weight, we measured the pups’ weight for three weeks after weaning. Though it is possible to start weighing pups from the time of birth, disturbance of the litter before weaning risks the possibility of the mother consuming her pups. Thus, we decided that it was safer to weigh the mice after weaning when the pups can be more independent from their mother.

The mice were weighed every three days for a duration of three weeks (Figure 2b). As expected, Nisch^−/−^ pups are significantly smaller than both Nisch^+/−^ and Nisch^+/+^ mice; however, an unanticipated finding was that Nisch^+/−^ mice were significantly larger than Nisch^+/+^ mice as well. This finding may suggest that there is some part of the Nisch protein that was present in the previous loss-of-function Nisch mouse model that is responsible for modulating the metabolic pathways. Although the exact mechanism is not known, one possibility is that the C terminus is responsible for modulating sensitivity to insulin receptor substrate (IRS) proteins [12].

### 2.3. Nischarin Regulates Cell Migration

**Figure 3:**
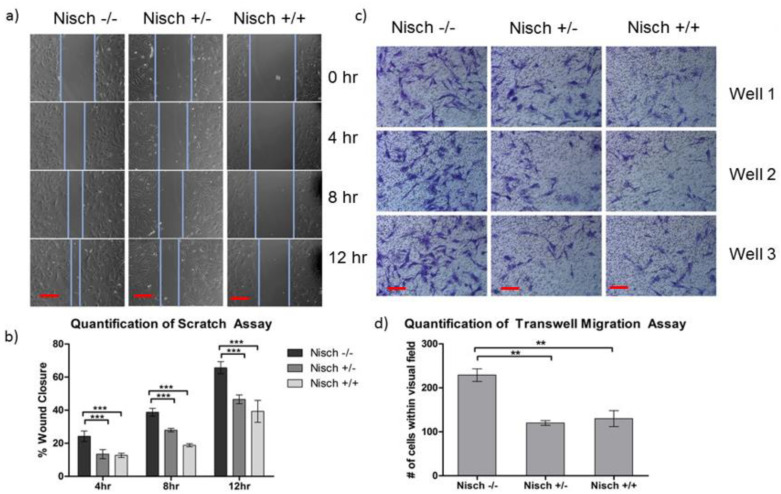
Nisch^−/−^ MEF cells demonstrate increased migration: (**a**,**b**) MEF cells were seeded in triplicate for each genotype and allowed to adhere to the plate for 16 h. A scratch was performed with the tip of a 200 µL pipette tip, and the cells were photographed at time points of 0, 4, 8, and 12 h. Nisch^−/−^ MEF cells demonstrated closure of the scratch at a faster rate than Nisch^+/−^ and Nisch^+/+^ cells; (**c**,**d**) MEF cells were seeded in transwell migration inserts in DMEM supplemented with 0.5% FBS. The bottom chamber was filled with DMEM supplemented with 10% FBS, acting as a chemoattractant for cells to travel through the pores. Nisch^−/−^ MEF cells had more cells migrate across the pores in the 16 h time period than Nisch^+/−^ and Nisch^+/+^ MEF cells. Statistically significant values of ** *p* < 0.01, and *** *p* < 0001 were assigned using two-way *t*-test analysis between the three genotypes. Experiments were performed in triplicate and repeated two times. Scale bar = 100 μm.

To corroborate the well-studied findings that Nisch plays an important role in cell migration, we conducted scratch and transwell assays to demonstrate the ability of Nisch to inhibit migration. MEF cells were cultured and seeded on the appropriate plates, as mentioned in the Methods Section. As expected, Nisch-null MEF cells demonstrated significantly increased migration, compared with heterozygous and wild-type Nisch cells, in both the wound healing assay (Figure 3a,b) and transwell assay (Figure 3c,d). Expression of Nisch was examined in multiple batches of Nisch Null, Het, and WT cells (Supplementary Figure S1). Even in normal cells, Nisch plays an important role in attenuating cellular movement. This supports the hypothesis that Nisch expression is indicated in neural cells during neurological maturation [2,5,6]. The ability of Nisch to inhibit migration is its best-defined characteristic. Several studies have confirmed that Nisch interacts with ITGA5, PAK1, LIMK1, LKB1, and Rac1 to inhibit cell motility [5]. To our surprise, cell proliferation was attenuated in Nisch null and Nisch Het cells, compared with Nisch WT cells (Supplementary Figure S2). It is not uncommon to have such an inverse relationship between cell proliferation and cell migration, as shown for P21CIP1 [13].

### 2.4. Nisch^−/−^ MEF Cells Demonstrate Altered Metabolism

**Figure 4:**
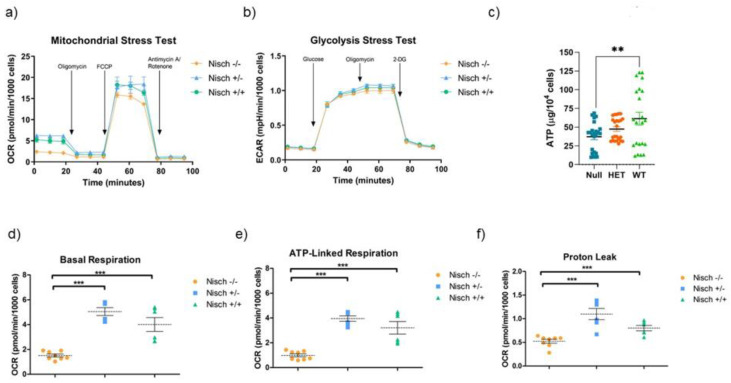
Real-time bioenergetic analysis reveals differences in mitochondrial activity. (**a**) Seahorse analysis was performed using the mitochondrial stress test conditions. Nisch^−/−^ MEF cells showed a significant decrease in basal respiration, ATP-linked respiration, and proton leak; (**b**) glycolysis stress test conditions were also tested. There were no significant differences between any of the parameters measured, indicating that the glycolytic pathways in cells of all three genotypes were not affected; (**c**) a total luminescence ATP assay revealed that Nisch-null MEF cells had an overall decrease in ATP levels, compared with wild-type MEF cells; (**d**–**f**) basal respiration, ATP-linked respiration, and proton leak parameters were examined closely to elucidate any trends. In each instance, Nisch-null MEF cells demonstrated a significant decrease in all of these parameters, while heterozygous and wild-type MEF cells did not exhibit any significant difference. Statistically significant values of ** *p* < 0.01, and *** *p* < 0001 were assigned using two-way ANOVA analysis between the three genotypes. Experiments were performed in triplicate and repeated two times.

To observe changes in metabolic profiles of Nisch^−/−^ mice, we conducted several assays using the Agilent Seahorse XF^e^-24 metabolic flux analyzer. We first performed a mitochondrial stress test and measured the oxygen consumption rate (OCR) (Figure 4a,d–f). The results show that Nisch^−/−^ cells had a lower basal respiration rate than Nisch^+/−^ and Nisch^+/+^ mice. Although there was no significant difference between the heterozygous and wild-type MEF cells, there was a trend suggesting that heterozygous mice had a slightly higher oxygen demand. As for ATP-linked respiration, Nisch^−/−^ demonstrated a much lower OCR than Nisch^+/−^ and Nisch^+/+^ MEF cells. These data represent the amount of oxygen consumed to drive ATP production. We hypothesize that the decrease in ATP-driven oxidative phosphorylation may be linked to higher rates of glycolysis.

A closer look at the Seahorse XF^e^-24 MST results shows that Nisch^−/−^ MEF cells had significantly less proton leak than Nisch^+/−^ and Nisch^+/+^ MEF cells. The amount of proton leak is typically associated with mitochondrial instability and increased reactive oxygen species (ROS) [14]. Lower proton leak in Nisch^−/−^ MEF cells suggests that there are some protective factors of the mitochondria in play, thus streamlining the ATP production process. To quantify the ATP production, we performed a total luminescence ATP assay and found that there was a significant reduction in ATP in the Nisch-null MEF cells, compared with wild-type MEF cells. (Figure 4c). The loss of one Nisch allele seemingly causes overcompensation of some metabolic pathways, resulting in mitochondrial instability and decreased efficacy in ATP production. However, the exact mechanism is currently unknown.

We also performed a glycolysis stress test (GST), which measures the extracellular acidification rate (ECAR) and provides information on the glycolytic activity of cells. Across all four parameters of glycolysis, glycolytic capacity, glycolytic reserve, and non-glycolytic acidification, Nisch^−/−^, Nisch^+/−^, and Nisch^+/+^ MEF cells all demonstrated comparable levels of activity, despite the findings that loss of Nisch is implicated to upregulate catabolic metabolism when it is not able to bind to AMPK [8]. The results for the GST are shown in Figure 4b.

**Figure 5:**
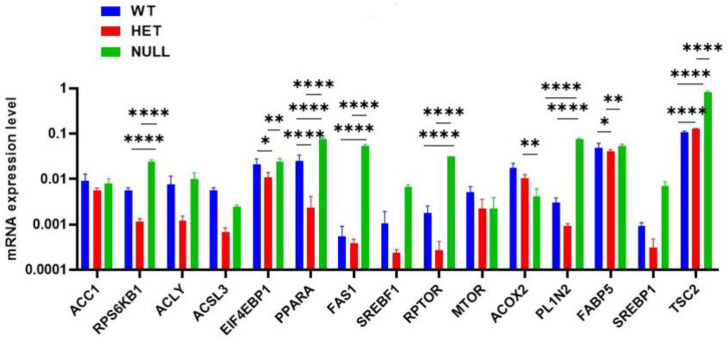
Real-time q-RT PCR analysis of various genes. Gene expression levels were normalized to GAPDH expression. Data are given as mean ± SD. Statistical analysis was performed using GraphPad prism 7 and a two-way ANOVA with Tukey’s multiple comparison post-test, comparing WT vs. HET or WT vs. NULL per condition. Significant differences are displayed: * *p* < 0.05, ** *p* < 0.01, and **** *p* < 0.0001 were determined, compared with the control. Experiments were performed in triplicate and repeated two times.

Finally, we sought to study metabolism by examining the changes in the expression of several genes that play important functions in lipid and fatty acid metabolism. Altogether, we decided to investigate 15 genes. Acetyl CoA carboxylase 1 (*ACC1*) catalyzes the carboxylation of acetyl CoA, thus forming malonyl CoA used in fatty acid synthesis [15]. ATP citrate lyase (*ACLY*) plays an important role in the synthesis of acetyl CoA from citrate [16]. Acyl CoA synthetase long-chain family member 3 (*ACSL3*) converts free long-chain fatty acids into fatty acyl CoA clusters that play important roles in lipid biosynthesis and fatty acid degradation [17]. Fatty acid synthetase (*FAS*) catalyzes the formation of long-chain fatty acids from acetyl CoA and malonyl CoA, thus involved in fatty acid metabolism [18]. Acyl CoA Oxidase 2 (*ACOX2*) oxidizes short- and long-chain 2-methyl branched fatty acids and, thus, is involved in fatty acid beta oxidation [19]. Fatty-acid-binding protein 5 (*FABP5*) binds to long-chain fatty acids and plays an important role in fatty acid uptake, transport, and metabolism [20]. In addition, we examined genes involved in cell growth. Ribosomal S6K B1 (*RPSRKB1*) is a serine–threonine kinase that responds to mammalian target of rapamycin (mTOR) signaling, to promote cell growth and proliferation [21]. We also looked at mTOR, which is a kinase composed of two subunits—mTORC1 regulates cell growth and proliferation, while mTORC2 regulates actin cytoskeleton and cell survival [22]. Next, EI4EBP1 directly interacts with eukaryotic translation initiation factor 4E (eIF4E). Phosphorylation of EI4EBP1 causes disassociation from eIF4E and promotes translation [23]. Peroxisome proliferator-activated receptor (*PPAR*) is a nuclear transcription factor important in lipid metabolism. This family of genes is also involved in cell differentiation. PPARα is known to regulate obesity by increasing hepatic fatty acid activation and decreasing the levels of triglycerides [24]. Sterol regulatory binding protein 1 (*SREBP1*) is a transcription factor that binds to the sterol regulatory element 1 (SRE1) and regulates the function of the low-density lipoprotein receptor gene [25]. The regulatory-associated protein of mTOR (*RPTOR*) forms complex with mTOR, as well as with eukaryotic initiation factor 4E binding protein and ribosomal protein kinase. It is involved in regulating the activity of mTOR, which regulates cell growth [22]. Perilipin 2 (*PLIN2*) belongs to perilipin family, which plays multiple roles in various cell types and has been shown to facilitate an important role in lipid accumulation in various diseases. The most well-studied function of this gene is in the development and maintenance of adipose tissue [26]. The *TSC2* gene, which codes for tuberin, is believed to be a tumor suppressor gene and can stimulate specific GTPases [27]. Tuberin is known to control cell growth and size [28]. Considering the importance of these genes in various metabolic and cell growth functions, we examined the expression pattern of these genes in cells from null, heterozygous, and wild-type Nisch mouse embryonic fibroblasts. As shown in Figure 5, expression levels of most of these genes are significantly enhanced in null cells, compared with WT and heterozygous cells. The changes were not significant for ACC1, ACLY, ACSL3, SREBP1, RPTOR, and mTOR, and the reasons for this are not known at this time, albeit the trend seems to be the same for all genes.

## 3. Discussion

In this study, we were able to confirm a complete knockout mouse model for Nischarin. Nisch^−/−^ mice exhibit an observable phenotype of being smaller. In monitoring the pups’ weight over the course of three weeks, we also inadvertently identified a phenotype for heterozygous mice, which are significantly heavier than wild-type mice. This finding establishes that the heterozygous expression of a protein can cause a significant increase in weight; however, further studies will need to be conducted to identify the metabolic cause of weight gain in the context of Nisch. Along the same lines, several studies reported that heterozygous conditions sometimes have advantages over wild-type conditions. For example, Klotho (KL) is a gene that plays an important role in Alzheimer’s disease. KL heterozygous condition protects aging-associated phenotypes and cognitive decline in AD patients, compared with the homozygous condition [29]. Furthermore, several new genomic studies depicted the importance of heterozygous conditions and suggested that the heterozygous condition needs to be evaluated with utmost care in the near future [30].

We then investigated the effect of Nisch on migration, which has been well documented in previous research [6]. Our results are consistent with the finding that Nisch plays a crucial role in inhibiting migration. The effect that Nisch has on migration is important in the context of cancer and is the reason for the nomenclature of Nisch. Increased migration in Nisch^−/−^ cells suggests that cancer cells suppress Nisch activity in order to facilitate metastasis, leading to progression of the disease and poor prognosis for cancer patients [31]. With higher levels of migration and invasion, cancers can metastasize to other parts of the body. In breast cancer, the most frequent locations of metastasis are the lungs, liver, and bone. It is important to fully characterize other cellular behaviors in addition to migration, such as metabolism, because of their potential for therapeutic targeting in cancer. Our findings show that Nisch can also influence mesenchymal cells, which supports the idea that Nisch facilitates neuronal development [2]. Previous studies also address Nisch’s role in the embryological development of the gut and reproductive organs [10].

As for metabolic studies, we found that despite having lower basal levels of respiration, Nisch^−/−^ has a higher ATP production rate, but the total amount of ATP is lower. A report states that the ratio of ATP generated per molecule of oxygen (P/O) can be variable [32]. Additionally, they discussed that when we account for oxygen consumption at the whole-body or tissue-specific level, there can be significant variations in ATP yield due to factors such as the ratio of protons being pumped into the intermembrane space per unit of oxygen consumed by the electron transport complex, the slippage of the proton pumps, and the dissipation of electrochemical potential across the inner mitochondrial membrane. We presume that Nisch^−/−^ mice are able to produce less ATP with lower basal respiration because of the decrease in proton leak. This finding supports the idea that loss of Nisch leads to decreased oxygen demands and ATP production.

The only other time the Seahorse assay was performed to study Nisch was in the loss-of-function Nisch-mutated mice that have been crossed with the PyMT oncogene, which causes spontaneous mammary tumors in mice [10]. Mice were bred to obtain Nisch^−/−^ -PyMT and Nisch^+/+^-PyMT mice. These mice were observed until they formed mammary tumors, at which point they were sacrificed, and the primary tumor cells were cultured. Seahorse XF^e^-24 analysis was performed, and the results showed that Nisch^−/−^ -PyMT had a much higher OCR, opposite of what we found in this study. There are some possible explanations for this, one being that this study was conducted in tumor cells, and the other being that the homozygous mutation of Nisch in the former study is the deletion of exon 7–10 encoding the LRR region, and the immunoblotting show that smaller molecular weight protein is still remaining in the knockout mouse lysate.

With regard to the expression of genes, higher expression was observed in null cells. ACSLs are important in the activation of long-chain fatty acids [33]. Since Nisch regulates the expression of ACSL3, PPARα, and CHREBP, activation of PPARα/AMPK expression of Nisch may be a novel approach to inhibit obesity and hepatic steatosis, and thus, Nisch may play an important role in obesity. Activation of PPARα leads to activation of downstream enzymes important in fat metabolism and glucose homeostasis [34], which can help diabetic obese patients. Additionally, a study indicates that PPARα increases adiponectin secretion that leads to increased hepatic fatty acid oxidation, which inhibits obesity-induced fatty liver [35]. Another study reported that adiponectin activates AMPK and PPARα, which ameliorate obesity and hepatic steatosis [36]. These mechanisms are particularly important in the regulation of Nischarin in obesity. Nisch may also regulate cell migration via PPARα, as it is known to regulate cell migration [37]. Similarly, suppressing lipogenesis is an approach for antiobesity, as Nisch deletion enhances lipogenic transcription factors such as PPAR and SREBP [38], which suppress lipogenesis. Nisch deletion promotes cell migration, and it may be akin to FAS, which promotes cell migration in prostate cancer cells [39]. Alternatively, Nisch could regulate cell migration through *ACC*, [18] *ACOX* [40], *FABP5* [41], *RPS6KB1* [21], *SREBP1* [25], and *TSC2* [42] pathways, as their expressions are shown to increase cell migration. In summary, Nisch may regulate multiple biological processes such as cell migration, lipogenesis, and fatty acid metabolism, and the expression of Nisch may have an advantageous effect on these processes.

## 4. Materials and Methods

### 4.1. Acquiring and Backcrossing Knockout Mice

Nischarin knockout mice were obtained from the MRC Harwell Institute in Oxfordshire, UK, with embryonic stem cells (ESC) obtained from the European Conditional Mouse Mutagenesis International Knockout Mouse Consortium (EUCOMM-IKMC). These mice were generated using a “knockout-first-reporter tagged insertion allele” strategy, in which a promoter-driven cassette is established in an ESC line. The cassette encodes β-galactosidase, neomycin resistance, and several FRT and loxP sites, which can be utilized for several genotyping strategies. Mice with the complete cassette have the conditional allele and are referred to as “Nisch-Tm1a”. When crossed with a Cre recombinase, the neomycin resistance gene and Nischarin exons 5–6 are deleted, thus creating a knockout strain or “Nisch-Tm1b”. Only Nisch-Tm1b mice were used for data acquisition and analysis in this study, although Nisch-Tm1a colonies were also backcrossed and maintained for future studies. Upon arrival, heterozygous Nisch-Tm1b mice were crossed with C57BL/6 mice until F3 mice were obtained.

### 4.2. DNA Extraction and Polymerase Chain Reaction

Pups were reared by their mothers until 21 days of age. At weaning, tail clippings of 1 mm were collected and dissolved overnight at 50 °C, in a mixture of 0.5 mg/mL proteinase K and Jack’s Lab tail lysis buffer (1 M Tris pH 8.0, 5 M NaCl, 0.5 M EDTA pH 8.0, 10% SDS). DNA was extracted with chloroform, precipitated with isopropanol, and washed with 70% ethanol. After allowing the pellets to dry for approximately 15 min, the DNA was resuspended in 10 mM Tris–EDTA buffer solution at 50 °C for 10 min. PCR products were run on a 2% (*w/v*) agarose gel at 85 V for 30 min with a 100 bp ladder for reference (New England Biolabs Inc., Ipswich, MA, USA, catalog number N3231S).

### 4.3. Growth Curve Measurements

At 21 days of age, pups were weaned from their mother and weighed every 3 days until 45 days of age. Measurements were taken with a scale (OHAUS, Shanghai, China, product number 71142845). The scale was sprayed with Clidox for sterilization to keep the mice specific-pathogen free (SPE), per guidelines set by the Institutional Animal Care and Use Committee (IACUC). Mice were handled by the tail and set onto the scale; measurements were recorded during a period of time when they were still for >3 s. Nisch^−/−^, Nisch^+/−^, and Nisch^+/+^ mice were measured, and the data shown represent the mean ± SEM.

### 4.4. Quantification of mRNA

RNA was extracted with TRIzol reagent (Invitrogen, Carlsbad, CA, USA, catalog number 15596026) per the manufacturer’s instructions. cDNA was produced from the mRNA by using a High-Capacity cDNA Reverse Transcription Kit (Applied Biosystems, Waltham, MA, USA, catalog number 4368814, lot 00844743), according to the manufacturer’s instructions. Quantitative real-time PCR was performed with iTaq Universal SYBR Green Supermix (Bio-Rad Laboratories Inc., Hercules, CA, USA, catalog number 1725121, batch 64317344), using the protocol included in the kit. The samples were processed with the appropriate settings on the Quant Studio 3 (Applied Biosystems, catalog number A28567). Triplicate repeats of each Nisch^−/−^, Nisch^+/−^, and Nisch^+/+^ for each probe were performed. The target gene expression levels were normalized to glyceraldehyde 3-phosphate dehydrogenase (GAPDH) levels, measured in the same reaction.

### 4.5. Western Blotting

MEF cell lysate was processed with the organic phase leftover from the TRIzol protocol. The solution was incubated with isopropanol and centrifuged at 15,000× *g* for 15 min at 4 °C [43,44]. The supernatant was discarded, and the pellet was washed with 70% ethanol. The pellet was then resuspended in lysis buffer (20 mM EDTA, 140 mM NaCl, 5% SDS) and briefly sonicated before the addition of 6X Laemmli buffer and incubation on a 50 °C heating block for 10 min. The samples were electrophoresed on a 7.5% gel using SDS–PAGE and transferred onto a polyvinylidene fluoride (PVDF) membrane (Merck Millipore Ltd., Tullagreen, County Cork, Ireland, catalog number IPVH00010). Nisch (Santa Cruz, Dallas, TX, USA catalog number sc-374407) and vinculin (Sigma, St. Louis, MO, USA catalog number V4505) antibodies were used to detect protein expression.

### 4.6. Establishing Mouse Embryonic Fibroblast Cell Lines

Nisch^+/−^ mice were intercrossed and closely monitored for a copulation plug [45]. At 13.5 days postcoitus, the pregnant mouse was sacrificed using isoflurane (Henry Schein Animal Health, Dublin, Ireland, OH, catalog number 029405) in a closed system located under a fume hood. The embryos were carefully removed and placed in a cold phosphate-buffered solution (PBS). The head and liver were removed to prevent culturing of neural and hepatic cells. Each embryo was then minced with sterile razor blades, suspended in 1 mL 0.25% trypsin–EDTA and then transferred to a 12-well plate and incubated at 37 °C for 5 min. The reaction was inactivated by adding 200 µL of 10% fetal bovine serum (FBS); then, each suspension was transferred to a 15 mL tube and centrifuged for 5 min at 1000 rcf. The supernatant was carefully aspirated, and the pellets were resuspended in 3 mL of warmed Dulbecco’s modified Eagle medium (DMEM) supplemented with 20% FBS (*v/v*) and 2% penicillin–streptomycin solution (*v/v*). The suspension was then transferred to 30 mm plates and kept in an incubator at 37 °C and 5% CO_2._ The following day, the MEF cells were passaged onto 10 cm plates for expansion. For genotyping, 200 µL of each suspension during MEF cell isolation were reserved and dissolved in 300 µL of Jack’s Lab tail lysis buffer at 50 °C for 30 min. DNA was extracted and amplified according to the methods previously described.

### 4.7. Transwell Migration Assay

MEF cells were seeded in transwell polyester membrane cell culture inserts (Corning Inc., Corning, NY, USA, product number CLS3452) at a density of 50,000 cells/well. Each MEF cell line was seeded in triplicates, suspended in a DMEM supplemented with 0.5% FBS (*v/v*) and 1% P/S (*v/v*). The bottom chamber was filled with a DMEM supplemented with 10% FBS (*v/v*) and 1% P/S (*v/v*). The cells were incubated in a heating chamber at 37 °C and 5% CO_2_ for 16 h. At the conclusion of this incubation period, the cells were then fixed in methanol and stained with crystal violet. Images were obtained at 20X with an Olympus IX81 microscope (Olympus America Inc., Melville, NY, USA) using CaptaVision imaging software (Accu-Scope, Commack, NY, USA).

### 4.8. In Vitro Wound Healing Assay

Cells were seeded in a 12-well plate in triplicates at a seeding density of 100,000 cells/well overnight [46]. At 70–80% confluency, cells were serum starved for 4 h. The cells were then replenished with complete media, and a scratch was performed with a 200 µL pipette tip. Photos at 10X were captured at 0, 4, 8, and 12 h with the Olympus IX81 microscope mentioned in previous methods, using Slidebook 5.0 (Intelligent Imaging Innovations, Denver, CO, USA). The percentage of wound closure was analyzed with ImageJ (https://imagej.nih.gov/ij/index.html, accessed on 1 December 2020).

### 4.9. Seahorse XF^e^24 Extracellular Flux Assay

Nisch^−/−^, Nisch ^+/−^, and Nisch^+/+^ MEF cells were tested for differences in metabolic phenotype using the mitochondrial stress test, glycolysis stress test, and ATP production rate assay. Cell number and drug concentrations were optimized before the experimental groups were used. In total, 40,000 cells were seeded onto Seahorse XF^e^24 cell culture microplates (Agilent, Santa Clara, CA, USA), and the assays were run according to the manufacturer’s protocols. The mitochondrial stress test (MST) measured oxygen consumption rate (OCR). Cells were treated with subsequent doses of 1 µM oligomycin (Sigma-Aldrich, St. Louise, MO, USA), 2.5 µM carbonyl cyanide-p-trifluoromethoxyphenylhydrazone (FCCP, Sigma-Aldrich), and a mixture of 0.5 µM rotenone, 1 µM antimycin A (both from Sigma-Aldrich), and 5 µM Hoechst dye (ThermoFisher, Waltham, MA, USA). The glycolysis stress test measured extracellular acidification rate (ECAR). Cells were treated with subsequent doses of 10 mM glucose (Sigma-Aldrich), 1 µM oligomycin, and a mixture of 50 mM 2-deoxy-D-glucose (2-DG, Sigma-Aldrich) and 5 µM Hoechst dye. Experiments were performed in 3–4 replicate wells for each MEF genotype. After completion of each assay, the microplates were placed inside a Cytation 5 (BioTek, Winooski, VT, USA) for fluorescent imaging and cell counting, made possible by the addition of Hoechst during the last treatment of drugs. The data were imported into the Seahorse Wave program, and the measurements were normalized to 1000 cells. Data were analyzed using the report generators provided by Agilent.

### 4.10. Estimation of ATP Levels in MEF

Cells were cultured as described above. Total ATP levels were tested using the ATPLite Bioluminescence assay system from PerkinElmer (Waltham, MA, USA), as described by the manufacturer. Briefly, 10,000 MEFs per well were resuspended in a 100 µL cell culture medium. Then, 50 µL of cell lysis buffer was added and shaken for 5 min at room temperature. As the next step, 50 µL substrate solution was added and shaken for 5 min. The plate with the cells was dark adapted for 10 min, and then, luminescence was measured in a luminescence plate reader (TriStar LB 941, Berthold Technologies, Oak Ridge, TN, USA). In parallel to the samples, an ATP standard was used, and a standard curve was plotted so that the absolute ATP concentration could be determined. Four different isolations of MEF were used, and each was measured in triplicates.

## Data Availability

All data provided in the manuscript. Any additional data will be provided upon request.

## Supplementary Materials

The following supporting information can be downloaded at: https://www.mdpi.com/article/10.3390/ijms23031374/s1.
