# Supplementary material for: Nischarin Deletion Reduces Oxidative Metabolism and Overall ATP: A Study Using a Novel NISCHΔ5-6 Knockout Mouse Model
Source: Int J Mol Sci. 2022 Jan 25;23(3):1374. doi: 10.3390/ijms23031374 (PMC8835720; doi:10.3390/ijms23031374)
Supplement: Supplementary file 1 [file ijms-23-01374-s001.zip › ijms-1422685-supplementary.pdf]

Supplementary Figure 1

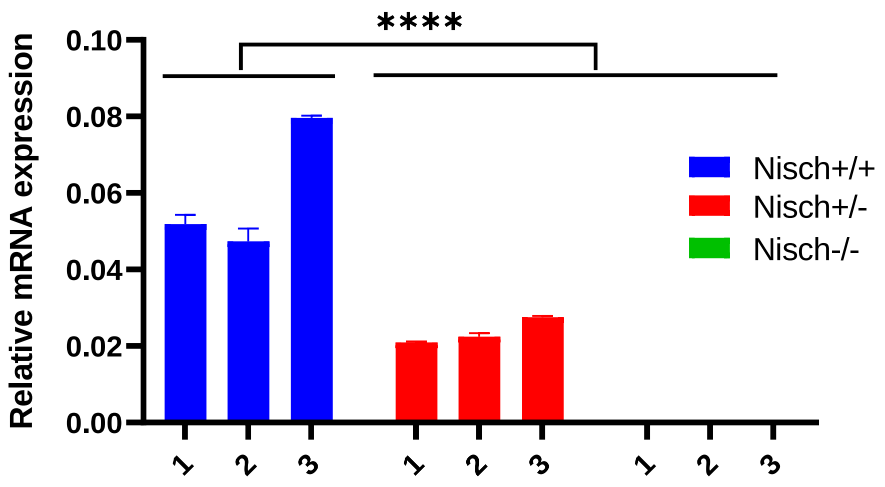

Total RNA was harvested for qRT-PCR analysis. Gene expression levels were normalized to *GAPDH*. Data are given as mean ± SD. Statistical analysis was performed using GraphPad Prism 7, using a two-way ANOVA with Tukey's multiple comparison post-test, comparing WT vs. HET or WT vs. NULL per concentration. Only significant differences are displayed: **p* < 0.05, ***p* < 0.01, and ****p* < 0.001 were determined compared with the control WTs.

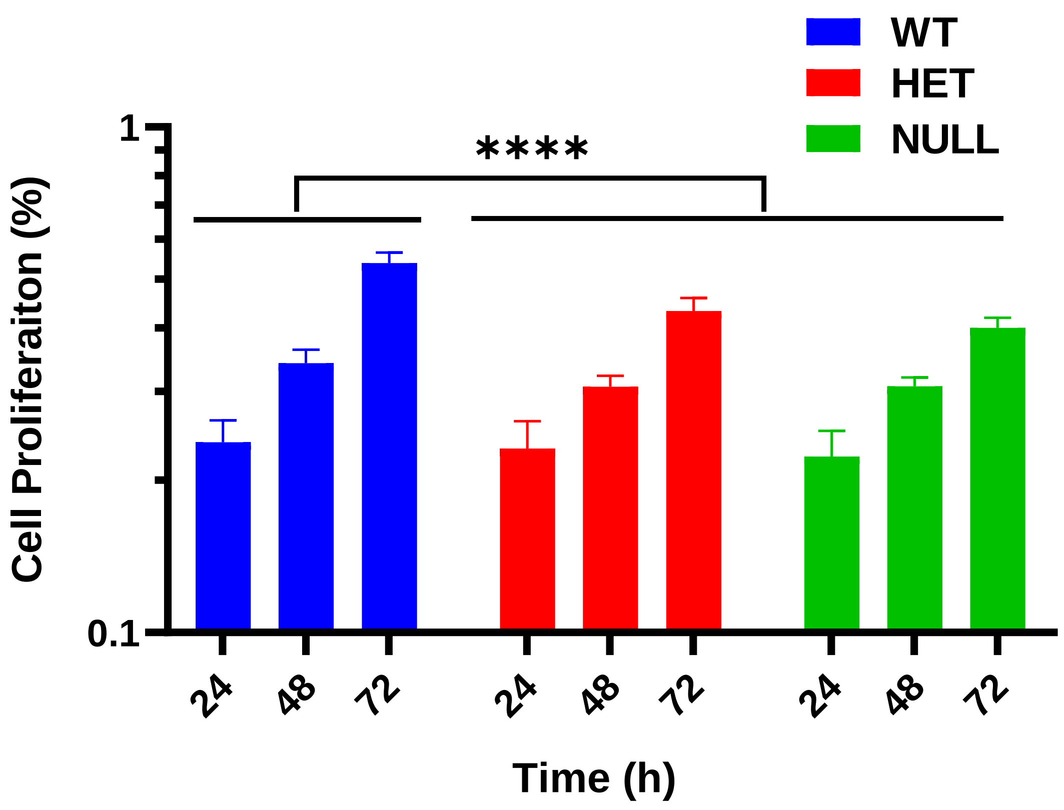

Supplementary Figure 2

Statistical analysis was performed using GraphPad Prism 7, using a two-way ANOVA with Tukey's multiple comparison post-test, comparing WT vs. HET or WT vs. NULL per concentration. Only significant differences are displayed: *$p < 0.05$, **$p < 0.01$, and ***$p < 0.001$ were determined compared with the control WTs.
